# How important is income in explaining individuals having forgone healthcare due to cost-sharing payments? Results from a mixed methods sequential explanatory study

**DOI:** 10.1186/s12913-022-07527-z

**Published:** 2022-02-15

**Authors:** Benjamin H. Salampessy, France R. M. Portrait, Marianne Donker, Ismail Ismail, Eric J. E. van der Hijden

**Affiliations:** 1 Department of Health Sciences, Faculty of Science, Vrije Universiteit Amsterdam, De Boelelaan 1085, 1081 HV Amsterdam, The Netherlands; 2 Zilveren Kruis (Achmea), Handelsweg 2, 3707 NH Zeist, The Netherlands

**Keywords:** Cost-sharing, Cost-related problems with access of healthcare, Mixed methods, Dominance analysis, Thematic analysis, Necessary care, Complexity of cost-sharing programs, Income, Financial leeway

## Abstract

**Background:**

Patients having forgone healthcare because of the costs involved has become more prevalent in recent years. Certain patient characteristics, such as income, are known to be associated with a stronger demand-response to cost-sharing. In this study, we first assess the relative importance of patient characteristics with regard to having forgone healthcare due to cost-sharing payments, and then employ qualitative methods in order to understand these findings better.

**Methods:**

Survey data was collected from a Dutch panel of regular users of healthcare. Logistic regression models and dominance analyses were performed to assess the relative importance of patient characteristics, i.e., personal characteristics, health, educational level, sense of mastery and financial situation. Semi-structured interviews (*n* = 5) were conducted with those who had forgone healthcare. The verbatim transcribed interviews were thematically analyzed.

**Results:**

Of the 7,339 respondents who completed the questionnaire, 1,048 respondents (14.3%) had forgone healthcare because of the deductible requirement. The regression model indicated that having a higher income reduced the odds of having forgone recommended healthcare due to the deductible (odds ratios of higher income categories relative to the lowest income category (reference): 0.29–0.49). However, dominance analyses revealed that financial leeway was more important than income: financial leeway contributed the most (34.8%) to the model’s overall McFadden’s pseudo-R2 (i.e., 0.123), followed by income (25.6%). Similar results were observed in stratified models and in population weighted models. Qualitative analyses distinguished four main themes that affected the patient’s decision whether to use healthcare: financial barriers, structural barriers related to the complex design of cost-sharing programs, individual considerations of the patient, and the perceived lack of control regarding treatment choices within a given treatment trajectory. Furthermore, “*having forgone healthcare”* seemed to have a negative connotation.

**Conclusion:**

Our findings show that financial leeway is more important than income with respect to having forgone recommended healthcare due to cost-sharing payments, and that other factors such as the perceived necessity of healthcare also matter. Our findings imply that solely adapting cost-sharing programs to income levels will only get one so far. Our study underlines the need for a broader perspective in the design of cost-sharing programs.

**Supplementary Information:**

The online version contains supplementary material available at 10.1186/s12913-022-07527-z.

## Background

Many countries have responded to the rising costs of healthcare by implementing some form of cost-sharing in which insured individuals pay part of the costs involved as an out-of-pocket (OOP) expense [1, 2]. Cost-sharing payments (also referred to as users’ fees or patient contributions) aim to increase the awareness of healthcare costs among those insured, but may also have counter-effects. Cost-sharing payments discourage insured individuals from seeking care. In turn, not seeking care can have adverse health effects [3]. Payments may consist of copayments (i.e., a fixed amount or a percentage of the costs per unit healthcare), deductibles (i.e., a predetermined amount paid by the individual after which the insurer covers all other costs) or a combination of both [2]. Rice et al. [4] have shown that OOP spending has risen or remained relatively high in many countries in the two last decades. In their analysis of high-income countries from the years 2000 onwards, they have observed that relatively high growth rates have been observed for those with historically low OOP spending (e.g., the Netherlands, France and the United Kingdom). Although smaller in relative growth rate, OOP spending has also increased among countries with historically high levels (e.g., the United States (US) and Switzerland) [4]. These trends have shifted a larger share of the costs to the insured individuals which forces them to devote an increasing portion of their annual income to these expenses [5, 6]. This shift should make those insured more aware of the costs involved which may, in turn, contribute to slowing down the rise of healthcare expenditures. However, this shift may also have "offset effects” as argued by Chandra et al. [7]. The authors have found that, while the rise of cost-sharing payments for outpatient physician visits and prescription drugs had resulted in a decline in the use of these services among older individuals, the number of hospitalizations increased. The authors have observed substantial offsets for the sickest populations with chronic conditions [7]. Hence, the policy shift towards more OOP spending may cancel out any costs initially saved due to cost-sharing if those insured forgo relatively cheap health services such as outpatient physician visits, but require additional and more expensive health services such as a hospitalization in the long run.

The rationale for implementing cost-sharing programs is underpinned by a large body of literature of which the RAND Health Insurance Experiment (RAND-HIE) has generated the methodological strongest evidence [1, 2]. The RAND-HIE has shown that cost-sharing payments reduce the demand for healthcare. The RAND-HIE has also revealed that this reduction occurs both in services with relatively little or no medical benefit for a patient’s health as judged by physicians (hereafter referred to as *non-recommended healthcare*), and in those with significant medical benefits (hereafter referred to as *recommended healthcare*) [8]. Hence, cost-sharing has often been described in literature as an effective yet blunt policy instrument [9–11]: for instance, Baicker and Goldman have argued that such payments create effective incentives that influence the demand for healthcare, but do so in indiscriminate manner with respect to recommended and non-recommended healthcare [11].

Besides studying OOP spending, Rice et al. [4] have also investigated perceived cost-related problems affecting access to healthcare using country-specific consumer survey data of Commonwealth Fund. In these surveys, respondents have been asked if they have forgone healthcare such as hospital visits and medication due to costs. The US and Switzerland (i.e., countries with high OOP spending) rank as the top two, while France ranks third despite its relatively low OOP spending: in 2016, 33%, 22% and 17% of the respondents respectively, had forgone healthcare due to costs [4, 12].

As certain individuals are more likely to struggle to afford rising cost-sharing payments, they are more prone to forgo healthcare because of these expenses [4, 5]. For instance, previous research suggests that low-income groups are more price sensitive than those with a high income [11]. The RAND-HIE have shown that the reduction of healthcare utilization due to cost-sharing have led to adverse health effects for those with the lowest income and in poor initial health [8]. Not using recommended healthcare in particular may result in the "offset effects” as described above [7].

Besides income, other patient characteristics might also play role and may even interplay with income, i.e., reinforcing or neutralizing each other’s effects. For example, on average and relative to high-income groups, low-income groups may be *more* likely to forgo healthcare due to costs [3]. However, among those with a low income, the amount of money available for discretionary spending may vary. Those with the smallest amount may be more likely to cut back healthcare due to costs than those with a larger amount of money available for discretionary spending. Hence, having more financial leeway may compensate for the effect of having a low income on the access to healthcare.

This study aims at gaining insights into the extent to which income explains individuals having forgone healthcare due to cost-sharing payments. Following a mixed methods sequential explanatory study design [13], we first use quantitative data to assess the relative importance of income and other patient characteristics with respect to having forgone healthcare due to cost-sharing payments. We then employ qualitative methods while applying an interpretative approach to understand better and enrich the quantitative findings. Our insights may be used to inform policy makers who must carefully design cost-sharing programs in such a way that they reduce the use of non-recommended healthcare and stimulate that of recommended healthcare, while providing adequate financial protection to prevent impoverishment of vulnerable groups.

### Dutch context

In this study, we have focused on the Dutch health system that similar to, for example, the US, is characterized by a relatively high healthcare expenditure: in 2018, as share of Gross Domestic Product: 10.0% (NL) and 16.9% (US) [14]. In addition, the Dutch government has implemented provider competition [15–17]. The reform of 2006 aims to stimulate effective competition between providers on price and quality, and to encourage patient choice. An important characteristic of the Dutch health system is universal access; it allows insured individuals to use healthcare—covered by the compulsory basic health insurance package—across *all* hospitals. Health insurers are allowed to offer various health plans that cover the same basic package but with different conditions. Health insurers are obligated to accept all applications, are not allowed to differentiate the premium of a plan across individuals and are compensated by a risk equalization fund for differences in the risk profiles of their insured population. Insured individuals older than 18 years pay an income-dependent contribution—capped at a specific income and paid through the employer—to this fund and a flat-rate premium directly to their insurer, while all costs of those aged under 18 are paid by the government. Those with a low-income are compensated by a healthcare allowance. In 2015, approximately one-third of the Dutch population (36%) has received some allowance [15]. According to Vermeend and Van Boxtel, the overall Dutch healthcare financing remains considered to be regressive after the 2006 reform [18].

The content of the basic package is determined by the Ministry of Health. According to its guidelines, only services that are deemed necessary, effective, efficient and otherwise unaffordable for most citizens are covered. For most such services, the General Practitioner (GP) serves as gatekeeper, while a mandatory front-end deductible is applicable to all covered services with the exception of a specific few such as GP care [15–17].

Rice et al. [4] have described the share of individuals that have forgone healthcare due to costs in the Netherlands as relatively low compared to that of, for example, the US. However, the authors have observed a ‘*dramatic fluctuation*’ in this measure for the Netherlands over time [4]. In 2010, 6% of the Dutch respondents indicated they had forgone healthcare due to cost-sharing payments. In 2013, this number peaked at 22%, but then declined to 8% by 2016. Rice and coauthors ascribe this pattern to the introduction of the deductible in 2008 and its relatively fast year-to-year increases thereafter. While initially capped at 155 euros when implemented, the deductible amount gradually increased to 170 euros in 2011 (+ 9.7% in three years), then rapidly expanded to 220 euros in 2012 (+ 29.4%) and 350 euros in 2013 (+ 59.1%). In part due to political pressure, the deductible’s threshold increased with relatively small increments to 385 euros (+ 2.6% to + 4.2% per year) between 2014 to 2016 and has remained fixed since then [4, 15, 19–21].

## Methods

### Phase 1: Quantitative survey

#### Data collection

We used data collected by Salampessy et al. [22] and described in detail elsewhere. In short, an online questionnaire was distributed by email among panel members of the Dutch Patient Federation in March and April 2016. This panel consisted of regular users of healthcare (e.g., individuals with a chronic condition) who thus had been faced with cost-sharing requirements on a regular basis. Participation in the study was voluntary and any contributions were anonymized. Based on these conditions, approval by the ‘Dutch Medical Research Involving Human Subjects Act’ was not necessary. The questionnaire included, among others, questions regarding the characteristics of respondents and any forgone healthcare due to costs. It focused on three main types of healthcare services that were ordered or prescribed by the treating physician, covered by the basic health insurance package and subject to the front-end mandatory deductible: (1) medications, (2) diagnostic tests and (3) referrals to medical specialists for consultation or treatment (hereafter referred to as *specialist care*).

#### ***Variables***

As our main outcome, we used the occurrence of recommended healthcare forgone due to the deductible. Respondents were included in the group *“having forgone healthcare”* (coded as 1) if they had forgone one of the aforementioned types of services, and in the group *“having utilized healthcare”* (coded as 0) if otherwise. To ensure that the healthcare forgone was linked to the payment of the deductible, we used the following question in the questionnaire: “*Have you forgone any healthcare recommended by a physician in the past two years due to mandatory deductible payments?*”. Similarly, we ensured that the use of healthcare was linked to an actual payment of the deductible. To do so, we included only those in the group *“having utilized healthcare”* that had to pay – either in full or in part – their deductible for the use of the given healthcare service as some respondents may already have paid their maximum deductible thus making any subsequent use ‘free’ of charge.

We used the various patient characteristics as determinants. We included three variables that reflected personal characteristics: gender, age (mean-centered, i.e., centered at the sample’s mean age) and household situation (binary: “living alone” (reference) and “married or living together”). We used two variables to describe an individual’s health status: presence of any chronic conditions (binary: “zero chronic conditions” (reference) and “one or more conditions”) and subjective health (three categories that ranged from “(very) poor” to “(very) good”). In addition, we included an individual’s highest attained educational level (three categories that ranged from “low “ to “high”) and sense of mastery level. The latter has been defined by Pearlin and Schooler as “*the extent to which one regards one's life-chances as being under one's own control in contrast to being fatalistically ruled*” (p5) [23], and was included as a proxy for an individual’s ability to exercise control over his or her health state. To measure sense of mastery, the 7-statements Pearlin Mastery Scale Test was used. In this test, each statement (e.g., “I have little control of events that happen to me”) was scored on a five-point Likert scale. Subsequently, a total score was computed that ranged from 7 (low sense of mastery) to 35 (complete sense of mastery) [24]. Similar to age, sense of mastery was centered at the sample’s mean value. Furthermore, we included two variables that reflected an individual’s financial situation: monthly net household income and financial leeway. Income^1^ was measured using five categories in total [25]: four categories that ranged from “less than €2000 per month” to “more than €4000 per month”, while a fifth category (labelled as “not-disclosed”) could be used if respondents did not know or did not want to state their income. Financial leeway^2^ reflected an individual’s financial status in terms of incurring debts or saving money [26]. This variable was measured by four categories: three of them ranged from “incurring debts or using savings” to “saving money” and a fourth category “not-disclosed”.

#### Econometric analysis

Of the 7,921 respondents described by Salampessy et al. [22], we included 7,339 respondents in our analyses. We performed a logistic regression model in which we used the occurrence of recommended healthcare forgone due to the deductible as dependent variable and included all determinants as independent variables.

To determine the relative importance of each determinant, we conducted dominance analyses [27]; we expected several determinants to be correlated, e.g., income was expected to be positively correlated with health. Previous research has shown that standardized regression coefficients in a multiple linear regression analysis are frequently used in the literature to determine relative importance; a method that is appropriate on the condition that the predictors are not correlated [27, 28]. Alternatively, this method may lead to erroneous conclusions; it only captures the amount of the ‘unique’ (i.e., non-correlated) part of the explained variance by the given predictor, while the squares of the computed indices do not aggregate to the overall model fit statistic (i.e., coefficient of determination that is often referred to as the explained variance or R-squared (R2)). Dominance analyses take the correlations between predictors into account. This technique computes the predictor’s contribution to the overall R2 and compares these contributions across all possible subset models (i.e., different combinations) for the given set of predictors. For dominance analyses based on logistic regressions models, McFadden’s pseudo R2 (R2_mf_) is frequently used as a model fit statistic [27, 28].

Inadditional analyses, we repeated our models stratified by type of healthcare service. We also performed inverse probability weighted (IPW) models to make our findings more representative of the total Dutch population [29]. To compute the weights, we used iterative proportional fitting and ensured that the weighted marginal totals of our sample’s age, gender and educational level closely resembled those of the total population [30].

All models were estimated in R [31]. Dominance analyses were performed using the “dominanceanalysis” package and R2_mf_ as model fit statistic. To increase the internal validity of our findings, all models and dominance analyses were bootstrapped using 1000 bootstraps with replacement [32]. Iterative proportional fitting was performed using the “anesrake” package. Across all analyses, any missing values and “not-disclosed” categories were treated as separate categories and modelled by using dummy variables (i.e., similar to the other categories). Results were considered statistically significant if p-value < 0.05.

### Phase 2: Qualitative follow-up

#### Sample

Given that the answers to the questionnaire were anonymous, we included an additional question: respondents could enter their contact information if they wanted to participate, on voluntary basis, in a follow-up interview. The Dutch Patient Federation withheld any contact information (i.e., this was not disclosed to members of research team) and contacted eligible individuals.

We started with a stratified purposive sampling strategy [33]: which initially aimed to recruit a sample of individuals who had forgone healthcare and whose social economic status levels and financial situations ranged widely. Based on the preliminary findings, we narrowed our sampling strategy by focusing only on those who had either (1) a low social economic status or (2) just enough money to live on, or were incurring debts or using savings. In March 2017, ninety individuals were contacted by telephone for interviews and asked if they had forgone healthcare. After the first round of phone calls (n = 30), we noticed that all the individuals were hesitant and refused to participate on hearing the topic of the interview. In the following rounds, we therefore introduced the topic in more neutral terms: we asked whether individuals had ever not followed up on healthcare that had been prescribed, ordered or referred by a physician. By doing so, we tried to keep away from any potential negative connotations that “*having forgone healthcare*” may have had. Given our quantitative-dominant study design, we followed a multiple case study approach: we interviewed only a small number of participants and did not set out to achieve data saturation.

#### Data collection

We first developed an interview guide (see Additional file 1) based on the relevant literature and the findings of our quantitative phase. To enrich the quantitative findings, we broadened the scope of our study: we asked about the occasions in which interviewees had forgone healthcare in general (i.e., GP care, medication, diagnostic tests, specialist care, long-term care and home care) regardless of whether it had been prescribed or ordered by a physician, and whether its costs had played a role in their decision.

Two interviewers conducted the semi-structured guided interviews: MD was well-acquainted with qualitative methods, while a research intern was closely supervised by BS, MD and EH. Interviews were conducted face-to-face at the interviewee’s home, audiotaped and transcribed verbatim. At the start of each interview, we informed interviewees that participation was voluntary and that their answers would be anonymized before being used for academic publication. Based on Dutch ethical principles of research, approval by the ‘Dutch Medical Research Involving Human Subjects was not required. All interviewees signed a written informed consent. Afterwards, a written summary was sent to each interviewee who, if necessary, could correct and elaborate the summary (member check).

#### Qualitative data analysis

As we used an interpretative approach, we performed a thematic analysis to order data and organize coded data into themes with respect to the research question. We analyzed inductively (i.e., data-driven), but we purposely did not set out to aim of generating theory from our findings. For a better understanding of the data, data collection and analysis were performed iteratively, and the topic list was refined accordingly. To improve the dependability and confirmability of our findings [34], multiple members of the research team independently coded the data, while the coding was afterwards discussed to achieve consensus. In addition, we ensured that all distinguished themes were directly supported by verbatim data from the interviews. Analyses were performed in MAXQDA [35].

## Results

### Phase 1: Quantitative results

#### ***S***ample

Of the 7,339 respondents included in the main analysis (Table 1), 1048 respondents (14.3%) had forgone recommended healthcare due to the deductible (group: “*having forgone healthcare”*) and differed from those who did not (group: *“having utilized healthcare”*). On average and relative to the group *“having utilized healthcare”*, a larger share of the group “*having forgone healthcare*” was younger, female, had a poorer health level and a lower prevalence of chronic conditions, had attained a lower educational level and scored lower on the mastery scale, had a lower income and were incurring debts or using savings. Most respondents in the group *“having utilized healthcare”* had used prescribed medication, while the majority of the group “*having forgone healthcare*” had forgone ordered diagnostic tests. Additional tables regarding respondents’ characteristics are included in Additional file 2. Distributions of characteristics between both groups remained similar in samples stratified by type of heath service relative to total sample. On average and relative to the total Dutch population, the study sample was older, consisted of more females and had attained a higher educational level.

**Table 1:** Study population (quantitative phase)

Group:		“Having utilized healthcare” (n = 6291)	“Having forgone healthcare” (n = 1048)
Age (in years) ***	Mean (sd)	63.0 (10.9)	57.8 (10.9)
Gender (%) ***	Male	49.1	39.4
	Female	50.9	60.6
Household situation (%) ***	Living alone	71.2	61.7
	Married or living together	27.3	35.8
	Missing	1.5	2.5
Self-reported health (%) ***	(Very) poor	18.5	21.8
	Moderate	40.9	46.1
	(Very) good	40.6	32.1
Chronic conditions (%) ***	None	15.4	18.4
	One or more	84.6	81.6
Education level (%) ***	Low	22.8	26.4
	Moderate	30.2	34.0
	High	42.8	35.2
	Missing	4.2	4.4
Sense of mastery (Pearlin’s scale) ^A^ ***	Mean (sd)	22.6 (5.7)	20.5 (5.9)
Monthly net household income (%) ***	< €2000	34.9	63.0
	€2001-€3000	25.9	15.6
	€3001-€4000	13.6	5.0
	> €4000€	7.6	2.1
	Not-disclosed	18.0	14.3
Financial leeway (%) ***	Incurring debts or using savings	19.7	44.0
	Just enough to live on	32.2	36.0
	Saving money	46.3	18.5
	Not-disclosed	1.8	1.5
***Respondents per healthcare service***			
N of individuals (% of group)	Prescribed medications	5537 (88.0)	475 (45.3)
	Ordered diagnostic tests	4189 (66.6)	738 (70.4)
	Specialist care	3603 (57.3)	662 (63.2)

*Chi square tests and independent t-tests were used to identify systematic differences between both groups. A* = *measured by the Pearlin Mastery Scale Test in which the lowest possible summed score of 7 reflected a lacking sense of mastery, while the highest possible score of 35 reflected a complete sense of mastery *[24]

sd standard deviation. *** p-value < 0.05. *** p-value < *0.01*

#### Relative importance

The logistic regression model revealed several significant associations (Table 2). Regarding the determinants reflecting personal characteristics, age was negatively associated, i.e., a protective factor. Those older than the average aged respondent had lower odds of having forgone recommended healthcare due to the deductible (odds ratio, OR (95% confidence intervals, 95%CI): 0.97 (0.96–0.97)). With respect to determinants describing an individual’s health, relative to respondents in poor health, those in moderate health (OR (95%CI): 1.57 (1.30–1.91)) and those in good health (OR (95%CI): 1.49 (1.19–1.88)) had higher odds of having forgone recommended healthcare due to the deductible, i.e., risk factor. In contrast, respondents with a chronic condition had lower odds (OR (95%CI): 0.56 (0.46–0.70)) of demonstrating such decision behavior compared to those with no chronic condition. In addition, sense of mastery (mean-centered) was a protective factor: OR (95%CI): 0.96 (0.94–0.97). With regard to variables reflecting an individual’s financial situation, income and financial leeway were both protective factors: for example, respondents who were saving money had lower odds (OR (95%CI): 0.28 (0.23–0.35)) of having forgone recommended healthcare due to the deductible compared to those either incurring debts or using their savings.

**Table 2:** Results of logistic regression model

Analysis:		Logistic regression model
		OR (95%CI) ^C^
*Variables*		
Intercept		0.45 (0.33–0.62)
Age (in years) ^A^	*Mean centered*	0.97 (0.96–0.97)
Gender	Male *(reference)*	
	Female	1.03 (0.90–1.19)
Household situation	Living alone *(reference)*	
	Married or living together	0.89 (0.76–1.05)
	Missing	0.97 (0.57–1.54)
Self-reported health	(Very) poor *(reference)*	
	Moderate	1.57 (1.30–1.91)
	(Very) good	1.49 (1.19–1.88)
Chronic conditions	None *(reference)*	
	One or more	0.56 (0.46–0.70)
Education level	Low *(reference)*	
	Moderate	1.03 (0.86–1.24)
	High	1.20 (0.99–1.44)
	Missing	1.14 (0.74–1.64)
Sense of mastery (Pearlin’s scale) ^B^	*Mean centered*	0.96 (0.94–0.97)
Monthly net household income	< €2000 *(reference)*	
	€2001-€3000	0.49 (0.40–0.59)
	€3001-€4000	0.34 (0.24–0.46)
	> €4000	0.29 (0.18–0.44)
	Not-disclosed	0.54 (0.43–0.65)
Financial leeway	Incurring debts or using savings *(reference)*	
	Just enough to live on	0.56 (0.47–0.65)
	Saving money	0.28 (0.23–0.35)
	Not-disclosed	0.50 (0.25–0.82)
N of observations		7339
Model fit	Overall R2_mf_	0.123

*Dependent variable: “the occurrence of recommended healthcare forgone due to the deductible”, i.e., forgone either prescribed medications, ordered diagnostic tests or specialist care due to the deductible. A* = *centered at the total sample’s mean age: 62.2 years (standard deviation: 11.1). B* = *centered at the total sample’s mean score: 22.3 (standard deviation: 5.8). C* = *reflects bootstrapped confidence intervals*

*OR* *Odds ratio. R2*_*mf*_ = *McFadden’s pseudo R2. 95%CI* = *95% Confidence Intervals (lower bound – upper bound)*

**Fig. 1:**
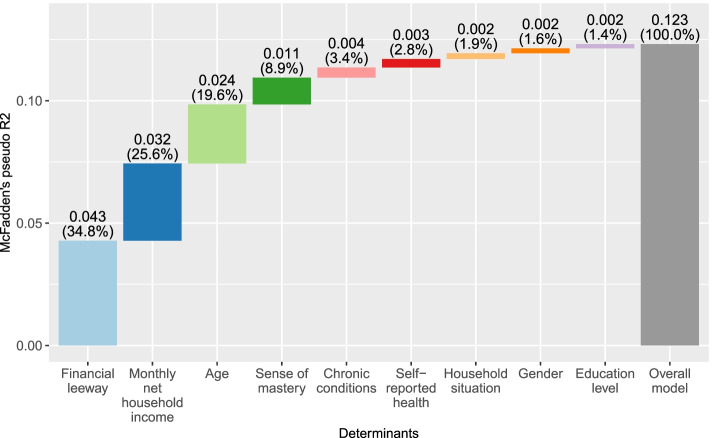
Results of dominance analysis. Values reflect the bootstrapped units of McFadden’s pseudo R2 of each determinant and its relative contribution to the model’s overall value

As shown in Fig. 1, dominance analysis revealed that financial leeway and income were the most important determinants as they contributed respectively 34.8% and 25.6% to the model’s overall R2_mf_ (i.e., 0.123). Together with age and sense of mastery, the four most important determinants contributed 88.9% to the aforementioned statistic.

#### Additional analyses

Results of additional analyses are included in Additional file 3. With regard to stratified models, results were similar to those of the main model: (1) significant associations remained significant except for self-reported health for prescribed medication. Among the significant associations, (2) the same sign was observed and (3) the ORs closely resembled those of the main model. (4) A similar contribution in the model’s overall R2_mf_ was observed for each of the four most important determinants, i.e., financial leeway, income, age and sense of mastery. With respect to IPW analyses, the model revealed similar results relative those of the unweighted model in terms of significant associations and corresponding direction, ORs and contribution in the model’s overall R2_mf_ with the one exception: self-reported health was not significant.

### Phase 2: Qualitative results

#### Sample

While contacting eligible individuals, it was noted that many of them declined the offer to participate as they did not consider themselves to be individuals who have forgone healthcare; the term “having forgone healthcare” was therefore perceived to have some negative connotation. Hence, the topic of the interview was reframed into more neutral terms. Afterwards, twelve individuals (*n* = 12) agreed to be interviewed of whom seven later reconsidered and either declined or were unable to participate due to a hospital admission.

Five individuals were interviewed (Table 3). The interviewees resembled, on average, the group “*having forgone healthcare*” in terms age, gender and health, but had attained a lower educational level, scored lower on the sense of mastery scale, had a lower monthly net household income and a smaller financial leeway relative to the aforementioned group. Furthermore, R1 was considered to be a deviant case: relative to the average respondent in the group “*having forgone healthcare*” and the other interviewees, R1 was older, scored higher on the sense of mastery scale and had a higher income and was able to save money.

**Table 3:** Study population (qualitative phase)

Interviewee	Age (years)	Gender	Self-reported health	Education level	Sense of mastery (Pearlin’s scale) ^A^	Monthly net household income	Financial leeway
R1	73	Male	(Very) good	High	24	€3001-€4000	Saving money
R2	67	Female	Moderate	Low	13	< €2000	Just enough to live on
R3	47	Male	(Very) good	Low	14	< €2000	Incurring debts or using savings
R4	59	Female	(Very) poor	Moderate	17	< €2000	Incurring debts or using savings
R5	52	Female	(Very) good	Low	20	< €2000	Just enough to live on

All individuals had one or more chronic conditions. A = measured by the Pearlin Mastery Scale Test in which the lowest possible summed score of 7 reflected a lacking sense of mastery, while the highest possible score of 35 reflected a complete sense of mastery [24]

#### Themes

As shown in Table 4, four main themes were distinguished that affected the patient’s decision whether to use healthcare: (1) financial barriers, (2) structural barriers related to the complex design of cost-sharing programs, (3) individual considerations of the patient and (4) the perceived lack of control regarding treatment choices within a given treatment trajectory.

**Table 4:** Themes and subthemes

Themes	Subthemes
*1. The financial barriers that affected the patient’s decision whether to use healthcare*	Coverage of the (additional) health insurance plan
	Amount of the cost-sharing or direct payment (in the case of a non-covered healthcare service)
*2. The structural barriers related to the complex design of cost-sharing programs that affected the patient’s decision whether to use healthcare*	Being unsure whether the healthcare service is covered by the basic health insurance package due to its complex design
	Being unsure whether cost-sharing payments are required and unable to determine the amount of any required payments in advance due to the complexity of the billing process
*3. The individual considerations of the patient that affected the patient’s decision whether to use healthcare*	Perceived medical necessity of healthcare
	Coping with their changed level of self-reliance due to a (chronic) condition
	Previous experiences with the physician, the healthcare service and the health insurer
	Fear of the consequences of the use of healthcare
	Travel time and parking availabilities
*4. The perceived lack of control regarding treatment choices within a given treatment trajectory that affected the patient’s decision whether to use healthcare*	Perceived compulsory use of health care as part of a treatment trajectory once the trajectory has started

Theme 1: The financial barriers that affected the patient’s decision whether to use healthcare.

The content (i.e., coverage) of the basic health insurance package or an additional health insurance plan, and any cost-sharing requirements (i.e., the amount of the payment) determined the costs of healthcare that, in turn, played a role for all interviewees. If interviewees had to pay, they indicated they were more likely to forgo the given healthcare service. However, relative to interviewees with a lower income or limited financial leeway, the costs of healthcare played a smaller role (i.e., less likely to forgo healthcare) for the respondent with a higher income and more financial leeway (R1).

(R5) “Although the GP disagreed, I postponed last year’s blood test until the next year as this test was quite expensive relative to the deductible.”

(R1) “I take the costs into account. As long as I can afford it, I do not mind paying for healthcare.”

Four interviewees used healthcare on a regular basis such that they had to have paid the deductible in full in the last years. As a consequence, having to pay the deductible in itself played a small role whether to use healthcare.

(R2) “I pay the mandatory deductible fully each year but arrange payment in monthly installments”.

Theme 2: The structural barriers related to the complex design of cost-sharing programs that affected the patient’s decision whether to use healthcare.

The design of the cost-sharing program *itself* played a role as it indirectly affected the costs of healthcare: due to the complexity of the program, three of the five interviewees were often unsure whether a given healthcare service was covered by the basic health insurance package and, if so, subject to cost-sharing. Two interviewees only discovered about the costs when they had received the bill. They indicated that, if they had known about these costs in advance, they sometimes would not have used the healthcare service. On other occasions, two interviewees had forgone the given service beforehand as they were unable to determine whether costs would be reimbursed by their insurer and could not afford it otherwise. In contrast to the other interviewees, relative to the other interviewees, R1 read the policy conditions of his insurance plan, actively sought additional information if necessary, and optimized the coverage for his medical use by switching between insurance plans.

*(R3) “I was not fully sure if the costs of a treatment in a specialized center would be covered as information from different sources contradicted each other. Therefore, I did not follow up on the referral as I would not be able to afford it.”*

*(R1) “My previous health insurance plan did not cover dental implants. I switched to a more expensive health insurance plan with additional benefits before receiving my dental implants. By doing so, my dental implants were fully covered.”*

Theme 3: The individual considerations of the patient that affected the patient’s decision whether to use healthcare.

The perceived medical need for healthcare was an important factor as all interviewees were of the opinion that they should not use more healthcare than necessary. After the GP had made the referral for a particular healthcare service, three interviewees indicated that they would then make their own judgement regarding its medical necessity. Only if they agreed, they would use the healthcare service. Alternatively, four interviewees would not use or decide to stop using the given service if the expected or perceived medical benefits were too small. Hence, the perceived medical necessity could act as either a protective factor or as a risk factor.

(R1) “GP referred me to a dietician for my elevated blood sugar levels. I did not follow up on the referral as I believed I could improve my diet myself.”

(R5) “If my back issues arise, I would first wait and see whether the pain passes. I would only visit the GP if I believe that it is truly necessary.”

Moreover, three interviewees showed signs of having to learn to cope with being less self-reliant and that they had to learn how to accept their need for regular use of healthcare in order to live with their chronic conditions.

(R4) “I have a wheelchair and a guide dog. It is not because I like to have them, but because I need them to be able to go somewhere.”

(R5) “Although I know from previous experiences that I need healthcare to manage my pain. As I feel that I am not ready to act, I do not seek healthcare.”

Previous experiences with the physician, the healthcare service or the health insurer also played a role for all interviewees and could act both as a protective factor and as a risk factor. For example, having a good patient-physician relationship encouraged interviewees to adhere to the prescribed therapy, and vice versa.

(R3) “I was reluctant to visit my former GP as he had once failed to detect my infection. I am very pleased with my new GP: I can contact him for all problems.”

Similar to *previous experiences*, fear played a role in different ways. Two interviewees feared that they might become resistant to certain antibiotics or addicted to pain relief medication. These fears led them to use a smaller amount or use such medication less frequently than prescribed by the physician. In contrast, the interviewees’ fear of cancer or recurrence of a tumor was a powerful incentive to use their medication as prescribed.

*(R4) “I frequently use antibiotics. Last year, I was hospitalized due to antibiotics-resistant bacteria. Without telling my physician, I decided it would be better if I stopped taking the antibiotics because I still need them to be able to work in the future.”*

*(R1) “Although my GP had concluded that the spot on my skin was not anything to worry about, I visited the dermatologist. Friends of mine also had spots on their skins which turned out to be cancerous.”*

For two interviewees, travel time and parking availabilities had played a role in their decision to forgo healthcare.

(*R4) “I did not always follow up on my rehabilitation appointments as it took me three hours including waiting time to get there by bus.”*

Theme 4: The perceived lack of control regarding treatment choices within a given treatment trajectory.

On some occasions, interviewees believed that choosing not to use healthcare was not an option once a treatment trajectory had started. Once a diagnosis had been made, interviewees had to undergo the full treatment trajectory consisting of diagnostic tests, treatments and physician visits. This made them often feel overwhelmed with intense emotions. In addition, as they were unable to oversee the full treatment trajectory due to its complexity, they perceived the use of health services within this trajectory as compulsory. Interviewees believed that they could not refuse parts of that trajectory. As a result, interviewees indicated to be less likely to follow up on referrals if they expected that it may result in a full treatment trajectory.

*(R2) “After being diagnosed with colon cancer, I received 23 external radiation therapy sessions, followed by three days of internal radiation therapy. However, the oncologist discovered another tumor. I immediately received another series of radiation therapy, surgery and chemotherapy. Being diagnosed with cancer twice in short amount of time, one simply has to survive all the treatments.”*

*(R3) “Over the years, I have received various tests and treatments in three different hospitals, but none of these treatments seemed to have helped. The last physician referred to another hospital. Luckily, the first appointment was canceled and the treatment never commenced: the fourth hospital did not call me back to set up a new appointment nor did I call them myself*.”

## Discussion

### Principal findings

In the quantitative phase, we assessed the relative importance of patient characteristics with regard to individuals having forgone recommended healthcare due to cost-sharing payments. The regression model indicated that having a higher income reduced the odds of having forgone recommended healthcare due to the deductible (ORs of higher income categories relative to the lowest income category (reference): 0.29–0.49). The model also revealed several significant relationships across various determinants. Being older, the presence of one or more chronic conditions, having a higher level of mastery and more financial leeway were all shown to be protective factors (i.e., decreased the odds of having forgone recommended healthcare due to the deductible), while having a moderate or good self-reported health showed to be a risk factor (i.e., increased the odds). Dominance analyses revealed that financial leeway was the most important patient characteristic: this determinant contributed the most (34.8%) to the model’s overall R2_mf_ (i.e., 0.123), followed by income (25.6%), age (19.6%) and sense of mastery (8.9%). Relative to the main model, the results of additional models stratified by type of healthcare service and of the population weighted models (i.e., IPW models) revealed no meaningful differences.

In the qualitative phase, we conducted interviews to understand and enrich the quantitative findings. Four main themes were distinguished that affected the patient’s decision whether to use healthcare: (1) financial barriers, (2) structural barriers related to the complex design of cost-sharing programs, (3) individual considerations of the patient, and (4) perceived lack of control regarding treatment choices within a given treatment trajectory. Furthermore, “*having forgone healthcare*” seemed to have some negative connotation as the topic of the interview had to be reframed using more neutral terms.

### Possible explanations and comparison with the literature

Our quantitative findings indicating the importance of financial leeway and income, correspond with previous studies that have linked factors such as the price of a given healthcare service, available household resources and income to the response in demand for healthcare [3, 36, 37]. Our quantitative findings also correspond with our qualitative findings as analyses distinguished financial factors as a relevant theme. In addition, and in line with literature [3, 8, 11, 38], we found a stronger response to cost-sharing among low-income interviewees relative to those with higher incomes.

Moreover, dominance analyses revealed that financial leeway was more important than income. On the one hand, this implies that an individual who is able to save some money for future health expenses despite having a low income, is less likely to forgo healthcare due to these expenses, and vice versa. On the other hand, this finding reflects the impact that unexpected expenses (e.g., due to multiple cost-sharing payments) or a sudden drop in income (e.g., being self-employed with no clients) may have on an individual’s financial situation and, in turn, they forgo healthcare due to the costs involved. These findings correspond with our qualitative findings. As regular users of healthcare, interviewees often had to pay the full deductible. To minimize the impact of paying such deductibles on their financial leeway, most interviewees had arranged to pay by monthly installments. Having to pay the deductible in *itself* therefore played a minor role.

In line with literature [37, 39], our qualitative analyses distinguished the complexity of cost-sharing programs as a relevant theme, and also indicated that its relevance differed across educational level. Interviewees with a low to moderate educational levels had more difficulty in determining in advance whether, and if so, how much they had to pay for a given healthcare service. Unsure or unable to determine whether they could afford these costs, interviewees decided not to use the given healthcare service or stopped any future use. In contrast, the interviewee with a higher educational level was able to navigate effectively within the insurance plan.

Among the remaining determinants, being older, having one or more chronic conditions and having a higher level of mastery were protective factors, while having a better self-reported health level was a risk factor. It is reasonable to assume that, given their age and previous experience with the use of healthcare, older individuals and those with chronic condition are more likely to be aware of the potential adverse effects -that having forgone recommended healthcare may result in- compared to those who are younger or who have no chronic condition. Hence, they may be keener on maintaining their current level of health and thus be more incentivized to use the healthcare as recommended.

Regarding sense of mastery, our findings are in line with the literature: previous research has linked higher levels of mastery to better health levels, and suggests that those with such high levels are more capable (1) of effectively managing their health-related problems and (2) of using coping strategies to deal with these problems [40]. This mechanism also supports our qualitative findings as some interviewees had forgone healthcare because they had difficulties accepting their chronic conditions and the resultant problems.

Regarding self-reported health, our qualitative findings may provide an explanation. If the perceived medical benefits were too small considering their health level, interviewees would not use the given healthcare service despite their physician’s judgement. More specifically, having a better state of health may reduce the perceived medical benefits of the given healthcare service that, in turn, leads individuals to forgo healthcare. This post-referral consideration may also explain why many eligible individuals have declined to participate in our interviews as they would not classify themselves as individuals who forgo healthcare. Previous research has indicated that the term “*having forgone healthcare*” is often perceived as stigmatizing as it suggests that the individual acted irresponsibly and thus should be blamed for not using healthcare [41]. Hence, the seemingly rational consideration that individuals give to the matter after being referred, contradicts the common opinion that those who forgo healthcare are irresponsible.

Furthermore, as treatment trajectories are generally comprised of multiple health services, interviewees often perceived the use of health services as part of this trajectory as compulsory. Consequently, they may reconsider to follow up on a referral if they believed this could lead to a full treatment trajectory. Our findings are in line with literature. Lippiett et al. have shown in their review that treatment trajectories for lung cancer have been described as demanding in terms impact on everyday life (e.g., frequent hospital visits) [42]. According to Sav et al., when patients perceive the burden of treatment as high, non-adherence to treatment is the most likely consequence to occur [43].

### Implications

Our findings have several implications. First, the observed importance of financial leeway indicates that solely adapting cost-sharing programs to income levels to prevent certain individuals from seeking recommended healthcare due to the costs involved (e.g., lower payments for low-income groups) will only get one so far. Individuals who are faced with multiple expenses due to frequent use of healthcare find that they are left with little financial leeway. To prevent such accumulation of expenses, policy makers need to adopt a broader perspective in which they consider *all* healthcare expenses that an individual may have at a given time and design their cost-sharing programs accordingly. Moreover, as cost-sharing payments reduce the demand for both recommended and non-recommended healthcare [8, 11], policy makers should follow the design principles of value-based health insurance that directly link these payments to the ‘*value’* of the given healthcare service [44, 45]. More specifically, healthcare services that yield high value (i.e., substantial medical benefits for a patient’s health relative to their costs) should be subject to lower or no cost-sharing payments, while those with little value should be levied with higher payments. Policy makers should also consider the administrative costs^3^ involved [46]. An all-payer claims processing data infrastructure, as implemented in the Netherlands, may help to limit administrative costs. In the Netherlands, all invoices between hospitals and payers are sent to and processed by a nation-wide system (i.e., VeCoZo). Processing individually adapted cost-sharing payments through the same nation-wide system should help to reduce the administrative burden.

Second, the relevance of the complexity of cost-sharing programs warrants additional efforts aimed at improving the transparency of these programs. For example, relative to a front-end deductible, flat-fee copayments paid at point of care offer individuals clear and immediate information on the required payments in advance; in a hypothetical decision context, Salampessy et al. [22] have demonstrated that such payments stimulate adherence to recommended healthcare.

Third, policy makers and physicians should be aware that various personal considerations and the perceived compulsory use of healthcare play a role in whether an individual uses healthcare. It underlines the importance of shared-decision making; a process that Elwyn et al. [47] have defined as “*an approach where clinicians and patients make decisions together using the best available evidence*” (p971). Policy measures that improve patient-centered care in clinical practice may help physicians to address these issues during consultations.

### Strengths and limitations

A strength of our study is the use of an explanatory sequential study design. The mix of quantitative and qualitative methods enhances the quality of our inferences and leads to a deeper understanding of our findings [48]. In addition, we followed principles of good practice in qualitative research [34]. For example, we sought feedback on the summary of the interview (member check) to improve the credibility of our findings. Also, we collected and analyzed data iteratively, and discussed the findings with multiple researchers; all of which improved the dependability and confirmability of our findings.

Certain limitations to our study should however be noted. With regard to the quantitative phase, our sample was not representative of the whole Dutch population. Although IPW models based on weighted representative sample in terms of age, gender and educational level produced similar results, we did not have population data for other relevant characteristics such as health and sense of mastery. However, as our sample consisted of regular users of healthcare who have faced cost-sharing payments, their observed responses may resemble their decision behavior in real-life settings more closely, which improves the internal validity of our findings.

With respect to the qualitative phase, we did not achieve data saturation due to the small number of interviews. Also, due to this small number we may have missed other relevant perspectives such as those of young people. Both aspects reduce the transferability and dependability of our findings [34]. While more interviews conducted among a wider sample is required to capture all relevant themes (i.e., a full-scale qualitative study) and achieve data saturation, we believe that our qualitative data is rich enough considering its explanatory purpose: most quantitative findings have been supported by one or more subthemes.

Furthermore, recent studies have demonstrated that factors related to the COVID-19 pandemic affect a patient’s decision to use health care: for example, Karacin et al. have shown that fear for COVID-19 has reduced the adherence to chemotherapy among patients with cancer [49]. However, as our data was collected prior to the COVID-19 pandemic, we could not consider the effects of this pandemic. It remains unclear to which extent a factor such as fear for COVID-19 would have affected our quantitative findings. Regarding our qualitative findings, we expect that fear for COVID-19 will be distinguished as an additional (sub)theme.

## Conclusions

Our findings show that financial leeway is more important than income with respect to having forgone recommended healthcare due to cost-sharing payments. Besides (1) financial barriers related to the health insurance plan, other factors such as (2) structural barriers related to the complex design of cost-sharing programs, (3) individual considerations of the patient and (4) the perceived lack of control regarding treatment choices within a given treatment trajectory, also play an important role. Our findings imply that, if cost-sharing programs focus solely on lowering these payments, they will only partly succeed in their goal of preventing certain individuals from seeking healthcare due to costs involved. Our study furthermore underlines the need for a broader perspective in the design of cost-sharing programs, the need to improve the transparency of these programs and the importance of shared-decision making.

## Supplementary Information

**Additional file 1. **

**Additional file 2. **

**Additional file 3. **

## Data Availability

The datasets used and/or analyzed during the current study are available from the corresponding author on reasonable request.

## Abbreviations

GP: General Practitioner
IPW: Inverse probability weighted
OOP: Out-of-pocket
OR: Odds ratio
RAND-HIE: RAND Health Insurance Experiment
R2: R-squared
R2_mf_: McFadden’s pseudo R2
SD: Standard deviation
US: United States
95%CI: 95% Confidence intervals

## Footnotes

1 In 2017, the Dutch modal monthly income was equal to approximately 2833 euros (gross) or 2145 euros (net) [25].

2 According to a five-yearly survey of Statistics Netherlands, the average Dutch household in the lowest income quintile spends a larger proportion of their annual income on housing, water and energy (39.6%), food and non-alcoholic drinks (12.0%), and alcoholic drinks and tobacco (4.1%) relative to those in the highest quintile (26.0%, 9.7% and 2.3%, respectively), while the opposite holds with respect to healthcare (1.1% (lowest quintile) versus 1.4% (highest quintile)) [26].

3 Based on their analyses of healthcare expenditures across eleven high-income countries, Papanicolas et al. conclude that administrative costs appear to be one of main drivers of the difference in overall cost between the US and the other countries [46].
