# Supplementary material for: How important is income in explaining individuals having forgone healthcare due to cost-sharing payments? Results from a mixed methods sequential explanatory study
Source: BMC Health Serv Res. 2022 Feb 15;22:208. doi: 10.1186/s12913-022-07527-z (PMC8848639; doi:10.1186/s12913-022-07527-z)
Supplement: Supplementary file 1 — Additional file 1. [file 12913_2022_7527_MOESM1_ESM.pdf]

Additional file 1

Interview guide

1. **Introduction** (prior to the start of the tape)

    a. Introduce the research project, the aim and the background of the study. Describe briefly your involvement and interest in research.

    b. Explain the conditions of the interview (oral and written information).

    c. If the interviewee has no further questions regarding the conditions, let him or her sign the informed consent.

    d. Start the tape.

2. Have you ever not used GP care (due to costs)?

    Prompts:

    a. Reasons for not using the healthcare (deductible, costs, time, travel time, work, fear, complications, relationship with GP/physician)

    b. Perceived expected health benefits

    c. Experience regarding not using the healthcare (in hindsight, would you do it again?)

3. Have you ever not used prescribed medication (due to costs)?

    In addition to the prompts listed under 2):

    a. Information on cost-sharing requirements (e.g., copayments) in advance

    b. Adverse effects of medication
    c. Fear of antibiotics-resistant
    d. Fear of addiction to pain-medication

4. Have you ever not used ordered diagnostic tests (due to costs)?

   In addition to the prompts listed under 2) and 3):

    a. Fear of the diagnosis of cancer
    b. Travel time and parking costs/availability

5. Have you ever not used referred specialist care (due to costs)?

   In addition to the prompts listed under 2) to 4):

    a. Information on coverage and costs of tertiary healthcare (specialized center) by his/her insurance plan

6. Have you ever not used referred long-term care or home care (due to costs)?

   In addition to the prompts listed under 2) to 5):

    a. Needs assessment by the governmental Care Needs Assessment Centre.

7. Last question: Are there any aspects you feel are important but we have not talked about yet?
8. Thank the interviewee and **stop the tape**.
