# Supplementary material for: How important is income in explaining individuals having forgone healthcare due to cost-sharing payments? Results from a mixed methods sequential explanatory study
Source: BMC Health Serv Res. 2022 Feb 15;22:208. doi: 10.1186/s12913-022-07527-z (PMC8848639; doi:10.1186/s12913-022-07527-z)
Supplement: Supplementary file 2 — Additional file 2. [file 12913_2022_7527_MOESM2_ESM.pdf]

Additional file 2

Study population, additional tables

Relative to the total sample (n=7339), the distributions of patient characteristics between individuals “*having utilized healthcare*” and *“having forgone healthcare”* did not differ across the sample as stratified by type of heath care service (Supplementary Table 1).

Supplementary Table 1. Study population, stratified by healthcare service

Healthcare service:		Prescribed medications		Ordered diagnostic tests		Specialist care	
Group:		"Having utilized healthcare" (n=5537)	"Having forgone healthcare" (n=475)	"Having utilized healthcare" (n=4189)	"Having forgone healthcare" (n=738)	"Having utilized healthcare" (n=3603)	"Having forgone healthcare" (n=662)
Age (in years)	Mean (sd)	63.1 (10.8)	57.6 (10.9)	62.9 (10.8)	57.6 (10.7)	63.0 (10.7)	58.0 (10.9)
Gender (%)	Male	49.7	42.3	47.8	40.1	48.8	39.6
	Female	50.3	57.7	52.2	59.9	51.2	60.4
Household situation (%)	Living alone	71.4	59.4	72.3	60.4	72.2	60.6
	Married or living together	27.1	38.1	26.3	36.9	26.4	36.5
	Missing	1.5	2.5	1.4	2.7	1.4	2.9
Self-reported health (%)	(Very) poor	18.7	31.8	18.2	19.9	19.5	22.8
	Moderate	41.7	45.5	40.8	48.5	42.3	47.1
	(Very) good	39.6	22.7	41.0	31.6	38.2	30.1
Chronic conditions (%)	None	13.9	12.8	15.4	20.1	13.9	19.2
	One or more	86.1	87.2	84.6	79.9	86.1	80.8
Education level (%)	Low	22.7	28.5	22.1	25.6	21.7	26.1
	Moderate	30.4	34.5	30.3	34.1	30.3	33.2
	High	42.8	33.2	43.7	36.4	44.1	36.2
	Missing	4.1	3.8	3.9	3.9	3.9	4.5

Supplementary Table 1. *(continued)*

Healthcare service:		Prescribed medications		Ordered diagnostic tests		Specialist care	
Group:		“Having utilized healthcare” (n=5537)	“Having forgone healthcare” (n=475)	“Having utilized healthcare” (n=4189)	“Having forgone healthcare” (n=738)	“Having utilized healthcare” (n=3603)	“Having forgone healthcare” (n=662)
Sense of mastery (Pearlin’s scale) [A]	Mean (sd)	22.6 (5.7)	19.5 (5.9)	22.8 (5.8)	20.6 (5.9)	22.4 (5.8)	20.2 (5.9)
Monthly net income (%)	< €2000	34.3	68.7	34.5	64.1	34.9	65.4
	€2001-€3000	26.5	14.5	26.2	14.1	26.0	14.8
	€3001-€4000	13.9	3.8	13.8	5.6	13.8	4.5
	>€4000€	7.5	0.8	7.8	2.4	7.9	2.0
	Not-disclosed	17.8	12.2	17.7	13.8	17.4	13.3
Financial leeway (%)	Incurring debts or using savings	19.6	52.6	19.4	43.9	20.6	46.5
	Just enough to live on	32.4	34.3	31.5	35.0	32.3	36.4
	Saving money	46.3	12.0	47.4	19.5	45.3	16.0
	Not-disclosed	1.7	1.1	1.7	1.6	1.8	1.1

A = measured by the Pearlin Mastery Scale Test in which the lowest possible summed score of 7 reflected a lacking sense of mastery, while the highest possible score of 35 reflected a complete sense of mastery [1].

SD = Standard deviation.

On average and relative to the total Dutch population, our sample was older, consisted of more females and had attained a higher educational level. No population data was available for other relevant characteristics such as health and sense of mastery (Supplementary Table 2).

Supplementary Table 2. Study population compared to the total Dutch population

Group:		Total sample (n=7339)	Total Dutch population [A]
Age (%)	18-49 years	15.3	50.7
	50-64 years	39.5	26.2
	65 years and older	45.2	23.1
Gender (%)	Male	47.7	49.6
	Female	52.3	50.4
Education level (%)	Low	23.3	32.3
	Moderate	30.8	37.7
	High	41.7	28.6
	Missing	4.2	1.4

A = source [2].

References

1. Pearlin LI, Menaghan EG, Lieberman MA, Mullan JT: **The stress process**. *J Health Soc Behav* 1981:337-356. doi:10.2307/2136676
2. Statistics Netherlands: **StatLine**. https://opendata.cbs.nl/statline/#/CBS/nl/ (2021). Accessed July, 2021
