# Supplementary material for: How important is income in explaining individuals having forgone healthcare due to cost-sharing payments? Results from a mixed methods sequential explanatory study
Source: BMC Health Serv Res. 2022 Feb 15;22:208. doi: 10.1186/s12913-022-07527-z (PMC8848639; doi:10.1186/s12913-022-07527-z)

Additional file 3

Results of stratified models

Results of regression models stratified by type of healthcare service (Supplementary Table 3) were similar to those of the main model with one exception. Self-reported health was significant in the main model, while this was not the case in the model for the prescribed medication. In addition, results of corresponding dominance analyses (Supplementary Figures 1-3) were similar to those of the main model: a similar contribution in the model's overall $R2_{mf}$ was observed for the four most important determinants, i.e., financial leeway, income, age and sense of mastery.

Supplementary Table 3**.** Results of logistic regression models, stratified by type of healthcare service

Healthcare service:		Prescribed medications OR (95%CI) [C]	Ordered diagnostic tests OR (95%CI) [C]	Specialist care OR (95%CI) [C]
Variables				
Intercept		0.43 (0.27-0.68)	0.48 (0.32-0.71)	0.63 (0.42-0.98)
Age (years) [A]	*Mean centered*	0.96 (0.96-0.97)	0.96 (0.95-0.97)	0.96 (0.96-0.97)
Gender	Male (reference)			
	Female	0.89 (0.72-1.10)	0.92 (0.77-1.09)	0.97 (0.81-1.18)
Household situation	Living alone (reference)			
	Married or living together	0.91 (0.73-1.13)	1.01 (0.83-1.22)	0.97 (0.78-1.19)
	Missing	0.99 (0.44-1.83)	1.10 (0.55-1.94)	1.29 (0.64-2.35)
Self-reported health	(Very) poor (reference)			
	Moderate	1.09 (0.85-1.41)	1.85 (1.45-2.37)	1.58 (1.23-2.03)
	(Very) good	0.85 (0.60-1.20)	1.58 (1.17-2.16)	1.48 (1.07-2.06)

Supplementary Table 3. *(continued)*

Healthcare service:		Prescribed medications OR (95%CI) [C]	Ordered diagnostic tests OR (95%CI) [C]	Specialist care OR (95%CI) [C]
Chronic conditions	None (reference)			
	One or more	0.55 (0.40-0.79)	0.51 (0.40-0.65)	0.44 (0.34-0.59)
Education level	Low (reference)			
	Moderate	0.96 (0.74-1.23)	0.99 (0.78-1.24)	0.93 (0.75-1.18)
	High	1.12 (0.85-1.51)	1.16 (0.93-1.44)	1.17 (0.93-1.50)
	Missing	0.93 (0.52-1.57)	1.09 (0.67-1.65)	1.22 (0.74-1.83)
Sense of mastery (Pearlin's scale) [B]	*Mean centered*	0.95 (0.93-0.97)	0.95 (0.94-0.97)	0.95 (0.93-0.97)
Monthly net income	< €2000 (reference)			
	€2001-€3000	0.47 (0.34-0.62)	0.44 (0.34-0.56)	0.45 (0.35-0.58)
	€3001-€4000	0.31 (0.16-0.47)	0.36 (0.23-0.50)	0.31 (0.19-0.46)
	>€4000€	0.15 (0.03-0.35)	0.33 (0.16-0.50)	0.27 (0.12-0.46)
	Not-disclosed	0.50 (0.36-0.66)	0.53 (0.40-0.68)	0.52 (0.39-0.69)
Financial leeway	Incurring debts or using savings (reference)			
	Just enough to live on	0.47 (0.38-0.58)	0.56 (0.46-0.68)	0.56 (0.46-0.69)
	Saving money	0.19 (0.14-0.26)	0.30 (0.23-0.38)	0.26 (0.20-0.34)
	Not-disclosed	0.35 (0.07-0.74)	0.59 (0.27-1.10)	0.36 (0.11-0.70)
N of observations		6012	4927	4265
Model fit	Overall $R2_{mf}$	0.15	0.13	0.14

Dependent variable: "the occurrence of prescribed medication / ordered diagnostic tests / referred specialist care forgone due to the deductible". A = centered at the total sample's mean age: 62.2 years (sd: 11.1). B = centered at the total sample's mean score: 22.3 (standard deviation: 5.8). C = reflects bootstrapped confidence intervals.

OR = Odds ratio. $R2_{mf}$ = McFadden's pseudo R2. 95%CI = 95% Confidence Intervals (lower bound-upper bound).

Supplementary Fig. 1 Results of dominance analysis (prescribed medications). Values reflect the bootstrapped units of McFadden's pseudo R2 of each determinant and its relative contribution to the model's overall value.

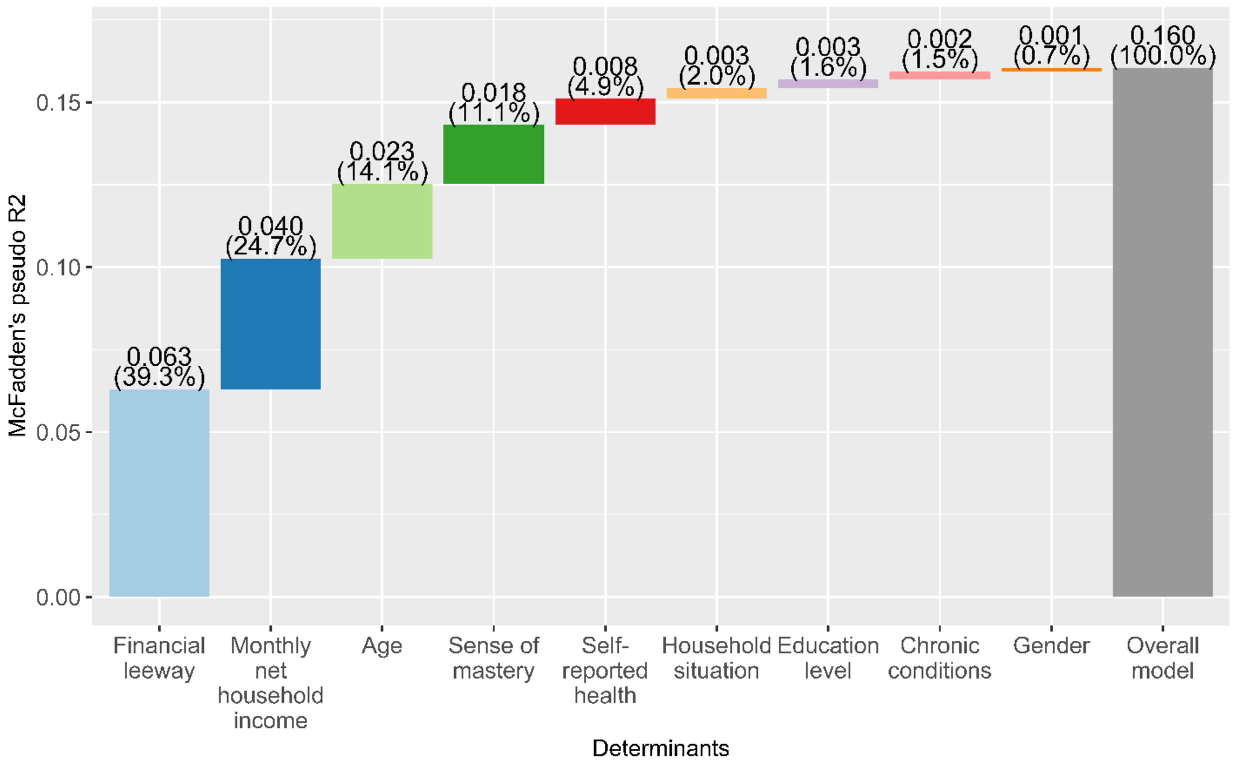

Supplementary Fig. 2. Results of dominance analysis (ordered diagnostic tests). Values reflect the bootstrapped units of McFadden's pseudo R2 of each determinant and its relative contribution to the model's overall value.

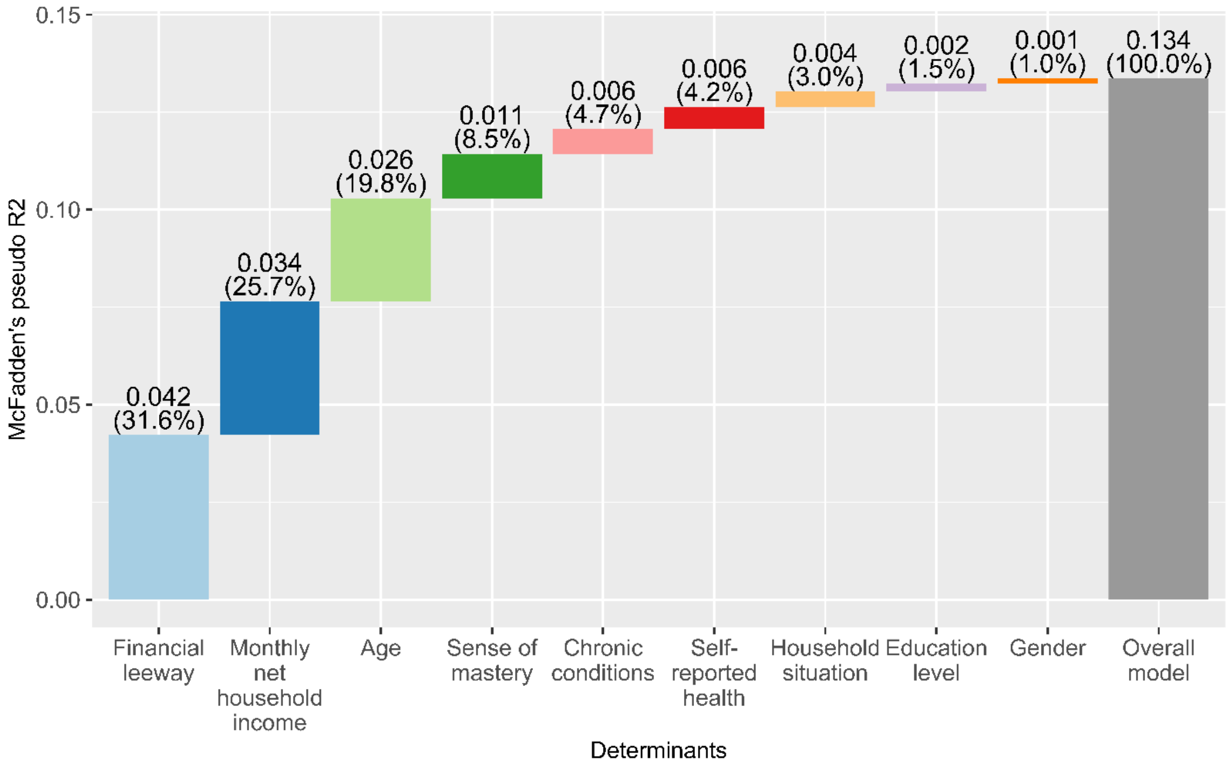

Supplementary Fig. 3. Results of dominance analysis (specialist care). Values reflect the bootstrapped units of McFadden's pseudo R2 of each determinant and its relative contribution to the model's overall value.

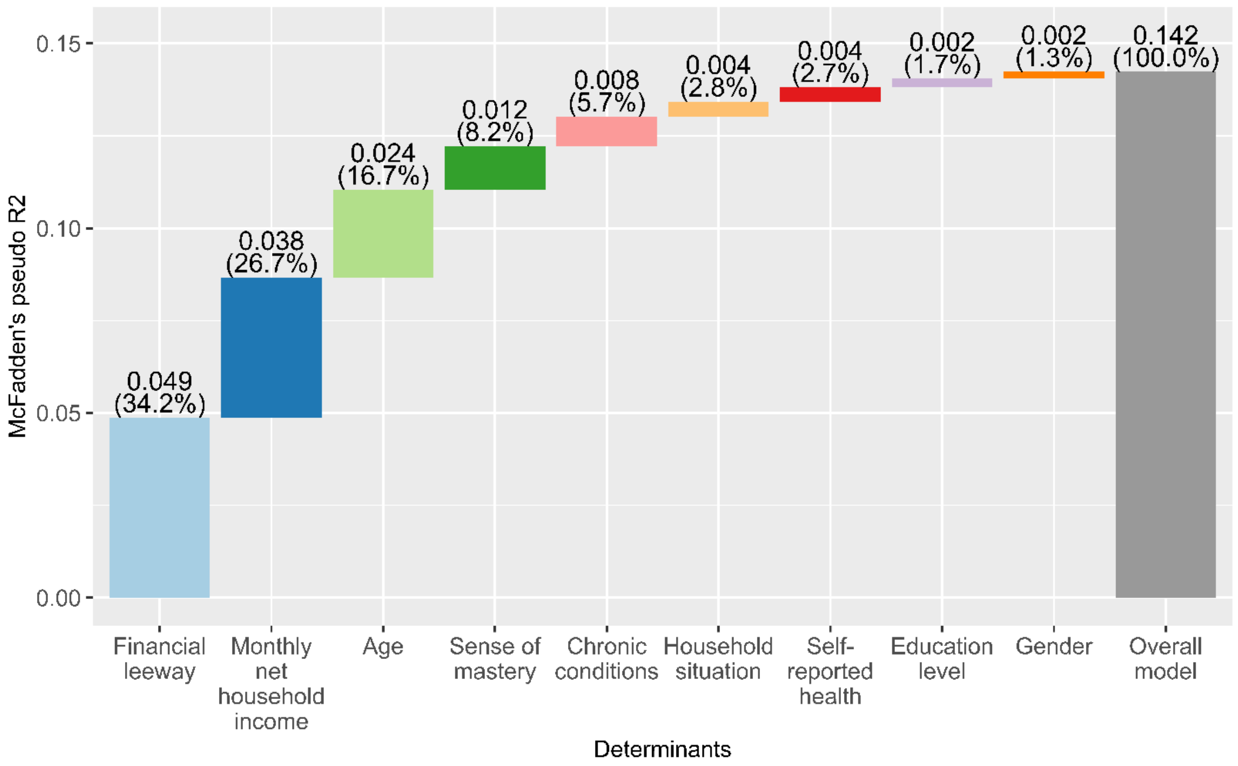

Results of population weighted (IPW) models

Results of population weighted regression model (Supplementary Table 4) resembled those of the main model except for self-reported health. Self-reported health was significant in the main model, while this was not the case in the model for the prescribed medication. In addition, results of corresponding dominance analysis (Supplementary Figure 4) were similar to those of the main model: a similar contribution in the model's overall $R2_{mf}$ was observed for the four most important determinants, i.e., financial leeway, income, age and sense of mastery.

Supplementary Table 4. Results of IPW logistic regression model

Analysis:		Population weighted logistic regression model<br>OR (95%CI) [C]
Variables		
Intercept		0.55 (0.34-0.88)
Age (in years) [A]	*Mean centered*	0.97 (0.96-0.98)
Gender	Male *(reference)*	
	Female	1.05 (0.85-1.32)
Household situation	Living alone *(reference)*	
	Married or living together	0.88 (0.70-1.13)
	Missing	0.81 (0.39-1.60)
Self-reported health	(Very) poor *(reference)*	
	Moderate	1.26 (0.97-1.65)
	(Very) good	1.18 (0.82-1.67)
Chronic conditions	None *(reference)*	
	One or more	0.59 (0.42-0.82)
Education level	Low *(reference)*	
	Moderate	0.97 (0.76-1.24)
	High	1.17 (0.88-1.52)
	Missing	1.29 (0.65-2.31)

Supplementary Table 4. *(continued)*

Analysis:		Population weighted logistic regression model<br>OR (95%CI) [C]
Sense of mastery (Pearlin's scale) [B]	*Mean centered*	0.97 (0.95-0.99)
Monthly net household income	< €2000 *(reference)*	
	€2001-€3000	0.44 (0.33-0.58)
	€3001-€4000	0.38 (0.22-0.60)
	>€4000	0.30 (0.13-0.55)
	Not-disclosed	0.57 (0.41-0.76)
Financial leeway	Incurring debts or using savings *(reference)*	
	Just enough to live on	0.52 (0.41-0.65)
	Saving money	0.28 (0.20-0.37)
	Not-disclosed	0.42 (0.16-0.85)
N of observations		7339
Model fit	Overall $R2_{mf}$	0.11

Dependent variable: "the occurrence of recommended healthcare forgone due to the deductible", i.e., forgone either prescribed medications, ordered diagnostic tests or specialist care due to the deductible. A = centered at the total sample's mean age: 62.2 years (standard deviation: 11.1). B = centered at the total sample's mean score: 22.3 (standard deviation: 5.8). C = reflects bootstrapped confidence intervals.

OR = Odds ratio. $R2_{mf}$ = McFadden's pseudo R2. 95%CI = 95% Confidence Intervals (lower bound – upper bound). IPW = Inversed probability weighted.

Supplementary Fig. 4. Results of dominance analysis (IPW). Values reflect the bootstrapped units of McFadden's pseudo R2 of each determinant and its relative contribution to the model's overall value.

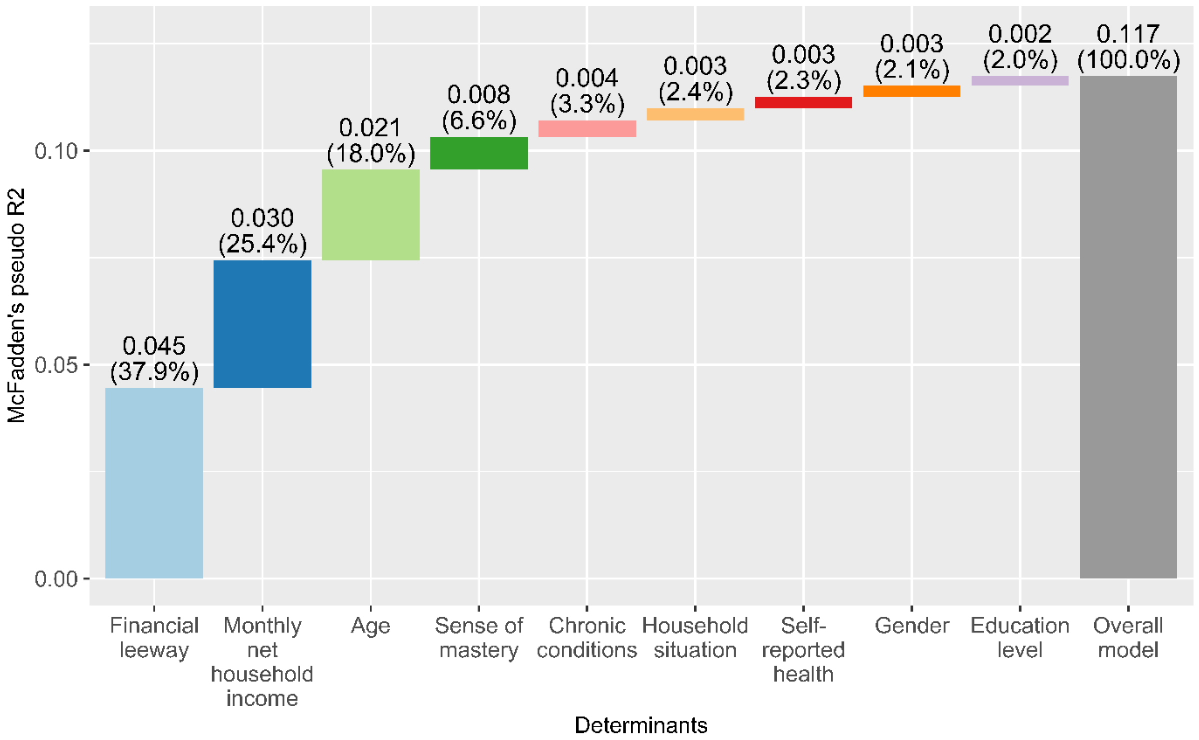

Supplement: Supplementary file 3 — Additional file 3. [file 12913_2022_7527_MOESM3_ESM.pdf]
